# Modifying the Electrocatalytic Selectivity of Oxidation Reactions with Ionic Liquids

**DOI:** 10.1002/anie.202202957

**Published:** 2022-05-24

**Authors:** Tian Yang, Juntao Yang, Xin Deng, Evanie Franz, Lukas Fromm, Nicola Taccardi, Zhi Liu, Andreas Görling, Peter Wasserscheid, Olaf Brummel, Jörg Libuda

**Affiliations:** ^1^ Interface Research and Catalysis FAU Erlangen-Nürnberg Germany; ^2^ School of Physical Science and Technology Shanghai Tech University China; ^3^ Lehrstuhl für Theoretische Chemie FAU Erlangen-Nürnberg Germany; ^4^ Lehrstuhl für Chemische Reaktionstechnik FAU Erlangen-Nürnberg Germany; ^5^ Helmholtz-Institut Erlangen-Nürnberg for Renewable Energy Germany

**Keywords:** Electrochemistry, Interphases, Ionic Liquids, Reaction Mechanisms, Solid Catalyst with Ionic Liquid Layer

## Abstract

The “solid catalyst with ionic liquid layer” (SCILL) is an extremely successful new concept in heterogeneous catalysis. The idea is to boost the selectivity of a catalyst by its modification with an ionic liquid (IL). Here, we show that it is possible to use the same concept in electrocatalysis for the selective transformation of organic compounds. We scrutinize the electrooxidation of 2,3‐butanediol, a reaction which yields two products, singly oxidized acetoin and doubly oxidized diacetyl. When adding the IL (1‐ethyl‐3‐methyl‐imidazolium trifluormethanesulfonate, [C_2_C_1_Im][OTf]), the selectivity for acetoin increases drastically. By in situ spectroscopy, we analyze the underlying mechanism: Specific adsorption of the IL anions suppresses the activation of water for the second oxidation step and, thus, enhances the selectivity for acetoin. Our study demonstrates the great potential of this approach for selective transformation of organic compounds.

## Graphical abstract

In electrocatalytic transformations of organic compounds, the selectivity can be controlled by adding an ionic liquid as a catalytic modifier. The concept is demonstrated for the selective oxidation of 2,3‐butanediol—a reaction which is of practical relevance for energy storage and the production of fine chemicals.

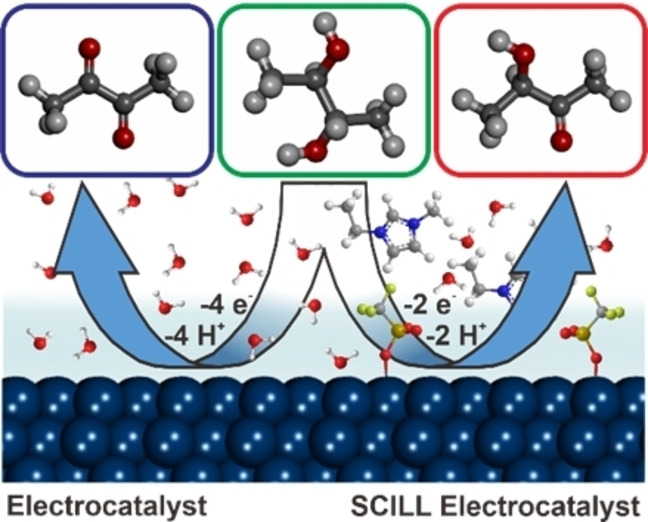

Improving selectivity is the major challenge in catalysis research.^[1]^ The selectivity of a catalytic process governs the materials efficiency, energy efficiency, and purification costs and, therefore, it is decisive for the economic competitiveness of a production process. In heterogeneous catalysis, a new concept has evolved recently to improve the selectivity: the so‐called “solid catalyst with ionic liquid layer” (SCILL).^[2]^ In a SCILL, the selectivity of a heterogeneous catalyst is modified by coating it with an ionic liquid (IL), which steers the reaction into the desired direction. The huge chemical versatility of ILs, often accompanied by high thermal and chemical stability,^[3]^ allows us to tailor the system to fit precisely the specific application. The SCILL concept is an outstanding success story as indicated by the recent release of the first commercial SCILL at industrial scale by Clariant AG.^[4]^

In heterogeneous catalysis, SCILLs are employed for the selective transformation of organic compounds. This raises the question whether we could use the same concept in electrocatalysis. Some groups showed that ILs can have a beneficial effect on activity in the electrocatalytic transformation of small molecules (such as the oxygen reduction reaction, ORR)^[5]^ The greatest potential of the SCILL concept lies, however, in a subtle control over the selectivity of organic compounds under mild conditions. This potential has not been explored in electrocatalysis so far. In this study, we show that we can indeed improve the selectivity of such an electrocatalytic transformation by adding an IL modifier. We provide the corresponding mechanistic insights.

Our test reaction is the selective electrooxidation of a dialcohol, namely 2,3‐butanediol (see Figure 1a) yielding two products, the singly oxidized acetoin and the doubly oxidized diacetyl. The selective oxidation of complex alcohols is highly relevant for two reasons. First, selective electrooxidation and reduction reactions enable the production of fine chemicals driven by electrical energy from renewable sources.^[6]^ Secondly, secondary alcohols can serve as rechargeable electrofuels to store renewable energy.^[7]^ In this work, we demonstrate that we can switch between the two reaction products by adding an IL. Secondly, we provide detailed mechanistic insights which explain our findings.

**Figure 1:**
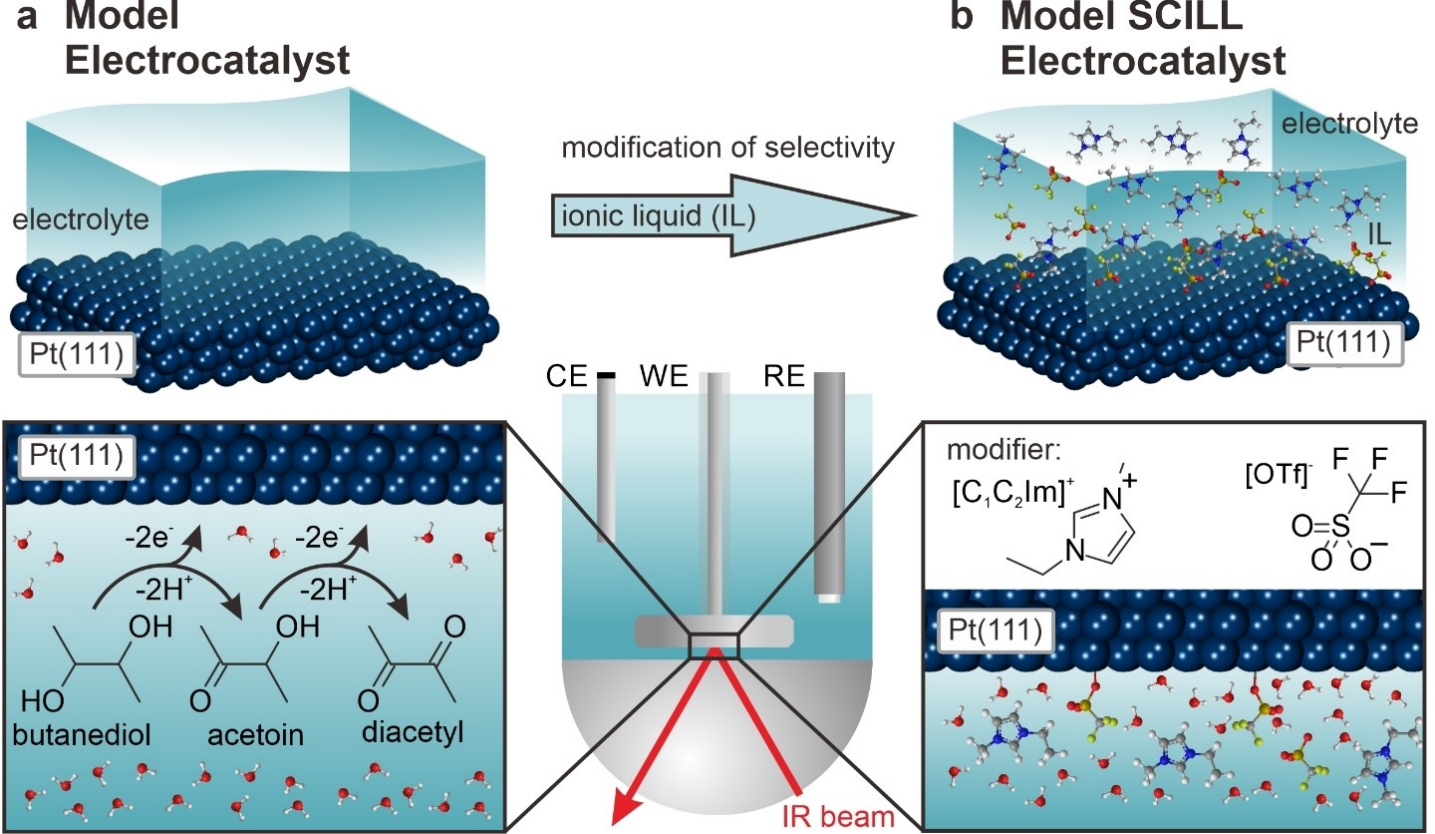
Catalysts and reactions studied in this work. a) Pt(111) single‐crystal electrode and oxidation of 2,3‐butanediol on Pt(111) in acidic electrolyte. Two products are formed: The acetoin with one alcohol function oxidized to the ketone and the diacetyl with both alcohol functions oxidized. b) Model system for the electrochemical SCILL: The ionic liquid [C_2_C_1_Im][OTf] is added as a modifier to the electrolyte and adsorbs on the Pt(111) surface.

Our strategy is inspired by the conceptual ideas first introduced by Jess et al.^[2a]^ and later studied by other groups as well.[^2b^, ^2c^, ^2d^, ^2e^, ^2f^] Briefly, the IL can affect the activity and selectivity in two ways: First, the solubility of reactants and products in the IL affects their concentration at the active site. Secondly, specific interactions of IL ions with the catalyst surface could change the selectivity by electronic or site blocking effects.[^2g^, ^2h^] Based on these considerations, we propose the following working hypothesis: For the electrocatalytic oxidation of alcohols, surface OH groups must be formed at the electrode surface. If the formation of surface OH is suppressed by strongly interacting ILs, the corresponding reaction will also be suppressed. If the blocking effect differs for two competing reactions, this will change the selectivity.

In this study, our catalytic modifier is the ionic liquid 1‐ethyl‐3‐methyl‐imidazolium trifluormethanesulfonate ([C_2_C_1_Im][OTf]) and the active electrode is an atomically‐defined Pt(111) single crystal (Figure 1b). We have chosen this IL, because the [OTf]^−^ anions adsorb specifically at Pt(111) in the potential range of interest.^[8]^ In addition, the IL is thermally^[9]^ and electrochemically stable^[3b]^ under operation conditions. The two partial oxidation reactions of butanediol occur at different electrode potential and the adsorption of [OTf]^−^ changes in between these potentials. Therefore, we expect that the IL has a strong influence on the selectivity of the electrocatalytic reaction. In this work, we prove that this is indeed the case. To this aim, we use in situ electrochemical infrared reflection absorption spectroscopy (EC‐IRRAS), which allows us to follow both the selectivity of the electrocatalytic reaction and the ion adsorption at the electrode interface.

In the first step, we studied the electrooxidation of butanediol by EC‐IRRAS in the absence of the IL (Figure 1a, Pt(111) electrode, 0.2 M 2,3‐butanediol, aqueous environment, 0.1 M HClO_4_, pH 1). We analyzed the IR spectra of the reactant (2,3‐butanediol) and the two reaction products (acetoin and diacetyl) by comparison with attenuated total reflection (ATR) IR spectra (Figure 2a) and simulated spectra from density functional theory (DFT). A detailed assignment of the bands is given in the Supporting Information (Section 2). For the present discussion, we focus on four bands only: The δ(CH_3_) band (1454–1458 cm^−1^) which is seen for butanediol and acetoin, the (ν_as_(CC)/δ(CH_3_)) band (1357–1362 cm^−1^) and the ν(C=O) band (1713–1715 cm^−1^) which are observed for acetoin and diacetyl and, very importantly, the ν(CC) band at 1200 cm^−1^, which is observed for the singly oxidized acetoin only.

**Figure 2:**
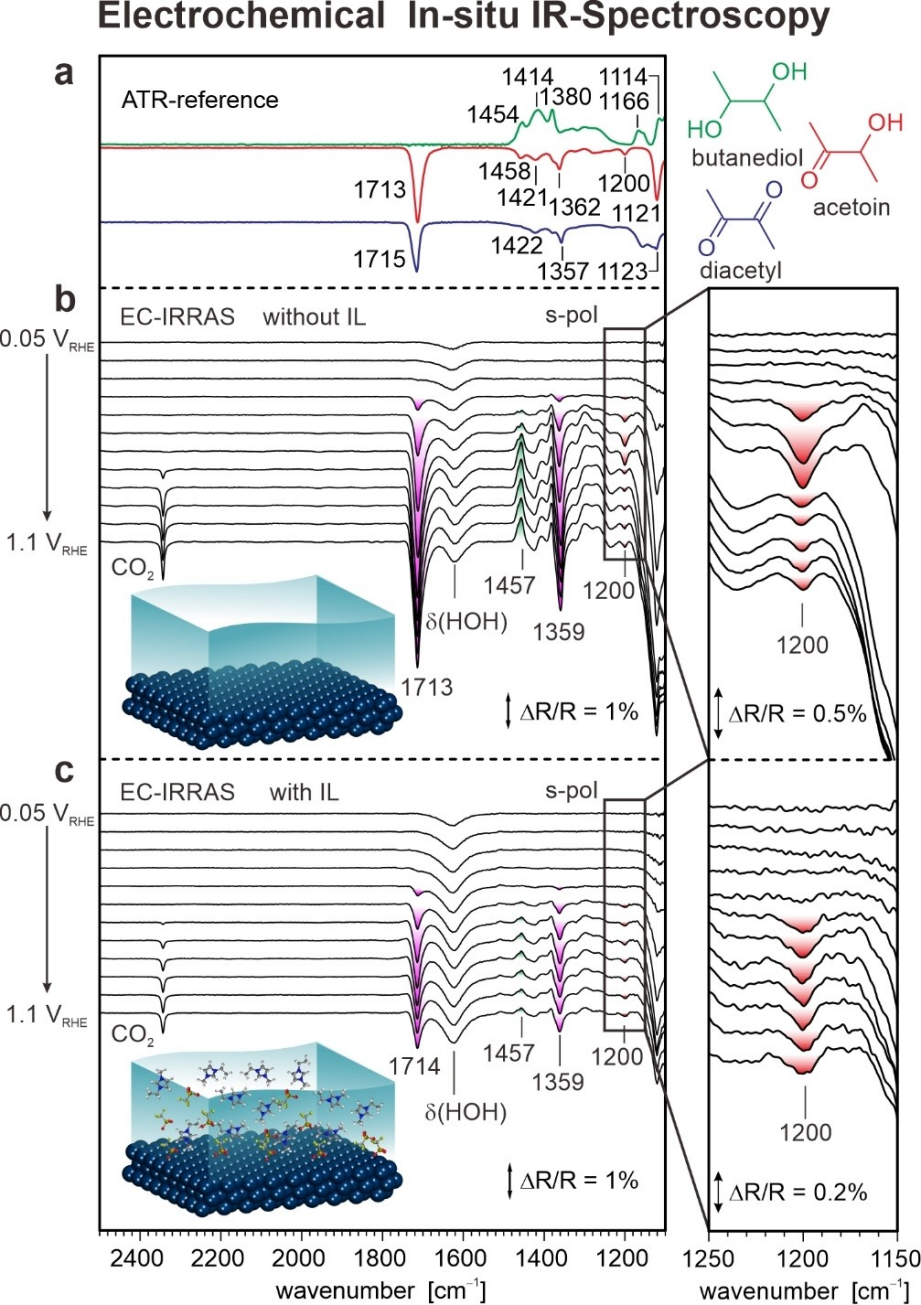
In situ IR spectra recorded during electrooxidation of 2,3‐butanediol in the absence and in the presence of the IL. a) Reference spectra of 2,3‐butanediol, acetoin, and diacetyl measured with ATR IR. b) In situ IR spectra recorded in the absence of the IL. c) In situ IR spectra recorded in the presence of the IL [C_2_C_1_Im][OTf] (1 mM). The band highlighted in red is characteristic for the partial oxidation product acetoin (see text for details). All spectra were measured with s‐polarized light. The reference spectra were taken at 0.05 V_RHE_.

We monitor the electrocatalytic transformation of butanediol to acetoin and diacetyl by in situ EC‐IRRAS in the potential region between 0.05 and 1.1 V_RHE_. The IR spectra in p‐polarization (see Supporting Information) and in s‐polarization provide information on the adsorbed species and the species in solution, respectively. In Figure 2b, we show the spectra recorded with s‐polarized light, which allow us to quantify the species in solution.^[10]^ In these difference spectra, positive bands (pointing upwards) indicate consumed species, while negative bands (pointing downwards) indicate species that are formed. Qualitatively, the positive band at 1457 cm^−1^ above 0.3 V_RHE_ indicates the consumption of butanediol, while the negative bands at 1713 cm^−1^ and 1359 cm^−1^ indicate the formation of acetoin and diacetyl. Very important is the band at 1200 cm^−1^ which is characteristic for the singly oxidized acetoin. The band intensity shows a maximum at 0.6 V_RHE_ and then decreases at higher potentials. We conclude that acetoin is initially formed with high selectivity and then further oxidized to diacetyl at potentials above 0.6 V_RHE_. These findings imply that the selectivity towards acetoin is limited by its further oxidation to diacetyl.

An additional weak band at 2343 cm^−1^ indicates the formation of very small amounts of CO_2_ as a byproduct. No other byproducts are detected. In particular, there is no adsorbed CO_ads_ formed in the reaction as shown by IR measurements with p‐polarized light (see Supporting Information). This finding is consistent with previous studies on secondary alcohols[^7b^, ^11^] and very important because CO_ads_ acts as a catalyst poison once it is formed.^[12]^

In the second step, we performed the reaction under identical conditions, however, we added a low concentration of the IL (0.001 M). Note that [C_2_C_1_Im][OTf] is soluble in water and stable in the potential region of interest.^[3b]^ The corresponding spectra are shown in Figure 2c. We observe the same bands as found in the absence of the IL, indicating consumption of butanediol and formation of acetoin and diacetyl. However, the band intensities are completely different. First, the intensity of the product bands (1714 cm^−1^, 1359 cm^−1^) is lower, indicating a decrease in activity. The most drastic change is observed in the behavior of the acetoin band at 1200 cm^−1^. While this band appears between 0.4 and 0.5 V_RHE_, there is no decrease at potentials above 0.6 V_RHE_ in sharp contrast to the IL‐free system. Qualitatively, this observation shows that the conversion of acetoin to diacetyl is blocked by the IL and, consequently, the selectivity to acetoin is enhanced.

In order to obtain quantitative information on the selectivity, we determined the concentrations of butanediol, acetoin and diacetyl from the IR spectra recorded with s‐polarized light (sensitive to species in solution, see Supporting Information for details). The results are shown in Figure 3a. The reaction sets in between 0.3 and 0.4 V_RHE_ both in the presence and in the absence of the IL. When we add the IL, the conversion drops from 38 % to 10 % indicating a loss of activity. This effect is accompanied, however, by a dramatic increase in selectivity to acetoin from 19 % to 56 % in the presence of the IL. We note at this point that a detailed kinetic analysis of the present data is complicated by the fact that the conversion and the reaction rates are changing simultaneously. Ideally, the initial selectivity should be recorded at each electrode potential, which was not possible, however, because of the detection limit of the experimental method applied. The fact that the two partial reactions show different onset potentials suggests, however, that it is possible to achieve true selectivity control via the IL.

**Figure 3:**
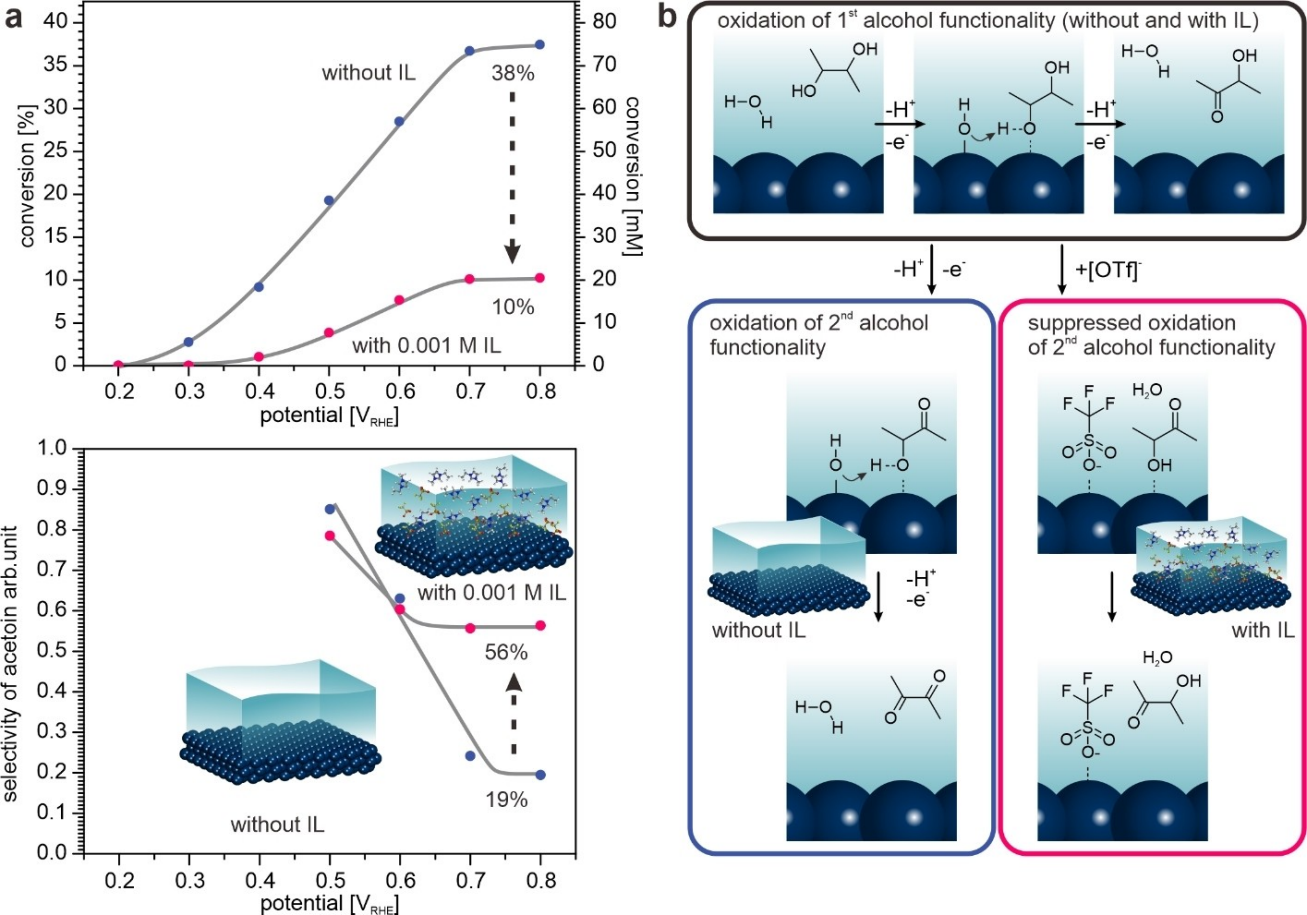
Effect of the IL on activity and selectivity as well as the proposed mechanism for selectivity control. a) Conversion of 2,3‐butanediol at the Pt(111) electrode and selectivity to acetoin as a function of the electrode potential in the absence and in the presence of the IL [C_2_C_1_Im][OTf] (1 mM) (see Supporting Information for details). b) Proposed mechanism for selectivity control in the electrochemical SCILL for selective oxidation.

We note that this type of behavior is very similar to SCILL systems in heterogeneous catalysis.^[2d]^ Here the activity often decreases in the presence of the IL, while the loss is accompanied by a greatly improved selectivity. For the technical process, the gain in selectivity is much more relevant than the loss in activity.

In order to prove our working hypothesis on the mechanism of selectivity control, we studied the potential‐dependent adsorption of the IL by in situ EC‐IRRAS under the relevant conditions (see Supporting Information). The IR data shows that the [OTf]^−^ anions adsorb specifically on Pt(111) in the potential window of interest while the surface concentration increases with increasing potential. The results are consistent with our previous work, in which we showed that the [OTf]^−^ ion adsorbs on Pt(111) via the SO_3_‐group in a tilted geometry partially suppressing the formation of OH species at the electrode surface.^[8]^

With these observations in mind, the effect of the IL modifier becomes clear: Co‐adsorption of OH at the electrode surface is necessary to oxidize the alcohol functionality (see Figure 3b).^[13]^ With increasing potential, the coverage of adsorbed [OTf]^−^ increases and, consequently, the surface concentration of OH decreases. As a result, the activity for the conversion to acetoin decreases as well. For the oxidation of acetoin to diacetyl the effect is, however, much larger, because this reaction requires a second OH group and occurs at higher potential, i.e. at even higher [OTf]^−^ coverage. As a result, the formation of diacetyl is very efficiently blocked and the selectivity to acetoin increases.

In summary, we have shown that it is possible to control the selectivity of the electrocatalytic transformation of an organic compound by adding an ionic liquid. For the selective oxidation of 2,3‐butanediol at a Pt(111) electrode we can switch between two products, acetoin and diacetyl, using [C_2_C_1_Im][OTf] as a catalytic modifier. We show that the effect originates from the specific adsorption of the [OTf]^−^ anion at the electrode surface in the potential region of interest. The [OTf]^−^ anion suppresses the formation of surface OH and, as a result, the conversion of acetoin to diacetyl. Consequently, the selectivity to the partial oxidation product acetoin increases drastically. Our study demonstrates the potential of EC‐SCILLs for selective electrooxidation and suggests a guiding principle for future development: Taking advantage of the huge chemical versatility of ILs, the interaction strength of the IL with the electrode should be tailored such that the degree of water activation is optimized in the relevant reaction window. We believe that this concept opens up new possibilities for tailoring selective electrocatalysts for energy conversion and chemical production. For future applications, it will be important to test this approach in real catalytic materials with a special focus on the long‐term stability of the ILs and the electrode materials.

## Conflict of interest

The authors declare no conflict of interest.

## Supporting information

As a service to our authors and readers, this journal provides supporting information supplied by the authors. Such materials are peer reviewed and may be re‐organized for online delivery, but are not copy‐edited or typeset. Technical support issues arising from supporting information (other than missing files) should be addressed to the authors.

Supporting Information

## Data Availability

The data that support the findings of this study are available from the corresponding author upon reasonable request.
