# Supplementary material for: Modifying the Electrocatalytic Selectivity of Oxidation Reactions with Ionic Liquids
Source: Angew Chem Int Ed Engl. 2022 May 24;61(29):e202202957. doi: 10.1002/anie.202202957 (PMC9400977; doi:10.1002/anie.202202957)
Supplement: Supplementary file 1 — Supporting Information [file ANIE-61-0-s001.pdf]

Angewandte Chemie
Eine Zeitschrift der Gesellschaft Deutscher Chemiker

Supporting Information

Modifying the Electrocatalytic Selectivity of Oxidation Reactions with Ionic Liquids

T. Yang, J. Yang, X. Deng, E. Franz, L. Fromm, N. Taccardi, Z. Liu, A. Görling, P. Wasserscheid, O. Brummel, J. Libuda**

Supporting Information

Modifying the Electrocatalytic Selectivity of Oxidation Reactions with Ionic Liquids

Tian Yang,[a,b] Juntao Yang,[a] Xin Deng,[a] Evanie Franz,[a] Lukas Fromm,[c] Nicola Taccardi,[d] Zhi Liu,[b] Andreas Görling,[c] Peter Wasserscheid,[d,e] Olaf Brummel,[a*] Jörg Libuda.[a**]

a) Interface Research and Catalysis, FAU Erlangen-Nürnberg, Germany

b) School of Physical Science and Technology, Shanghai Tech University, China

c) Lehrstuhl für Theoretische Chemie, FAU Erlangen-Nürnberg, Germany

d) Lehrstuhl für Chemische Reaktionstechnik, FAU Erlangen-Nürnberg, Germany

e) Helmholtz-Institut Erlangen-Nürnberg for Renewable Energy, Germany

*corresponding author: Dr. Olaf Brummel, olaf.brummel@fau.de

** also corresponding author: Prof. Dr. Joerg Libuda, joerg.libuda@fau.de

1. Adsorption of the Ionic Liquid on Pt(111)

By using subtractively normalized interfacial Fourier transform infrared reflection spectroscopy (SNIFTIRS), we investigate the adsorption behavior of 0.05 M [C_2C_1Im][OTf] in 0.1 M perchloric acid. Note that the acid was used as the supporting electrolyte in the butanediol oxidation experiment. For the SNIFTIRS experiment, we recorded reference spectra by using both s- and p-polarized radiation at 0.05 V_{RHE}, then the potential was stepped to a higher potential where the sample spectra were collected in both s- and p-polarization, see **Figure S1**. During the experiment, the potential of the Pt(111) working electrode switched between 0.05 V_{RHE} and the selected sample potentials. To correlate with the 2,3-butanediol oxidation experiment, we increase the sample potential from 0.1 to 1.1 V_{RHE} with a step of 0.1 V. The spectra at different sample potentials were calculated as (R_{sample}- $R_{reference}$)/$R_{reference}$ where the $R_{reference}$ is the preceding spectra at 0.05 V_{RHE}.

As shown in **Figure S2**, three negative bands appear in the frequency region between 1350 and 1150 cm^{-1} starting from 0.2 V_{RHE}, which are the characteristic IR bands for $[OTf]^-$ anion of the IL according to the literature.[1] Note that negative (pointing downwards) and positive (pointing upwards) bands indicate formed and consumed species, respectively. The peak at 1260 cm^{-1} is attributed to the asymmetric stretching vibration of the SO_3^- group. The peaks at 1228 and 1183 cm^{-1} are ascribed as the symmetric and asymmetric stretching vibration of the CF_3 group, respectively. A direct comparison of the spectra measured in p- and in s-polarization show that the bands are observable in p-polarization only. Note that EC-IRRAS bands observable in p-polarization only belong to adsorbed species due to the metal surface selection rule.[1] This confirms that the $[OTf]^-$ anion adsorbs specifically and potential dependent on the Pt(111) surface in the potential region of 2,3-butanediol oxidation. Note that we expect similar behavior also for other $[OTf]^-$ containing salts.

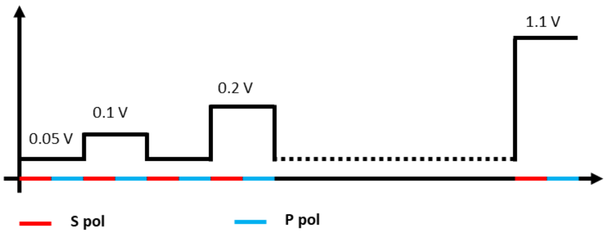

Figure S1. The procedure used in the SNIFTIRS experiment shown in **Figure S2**.

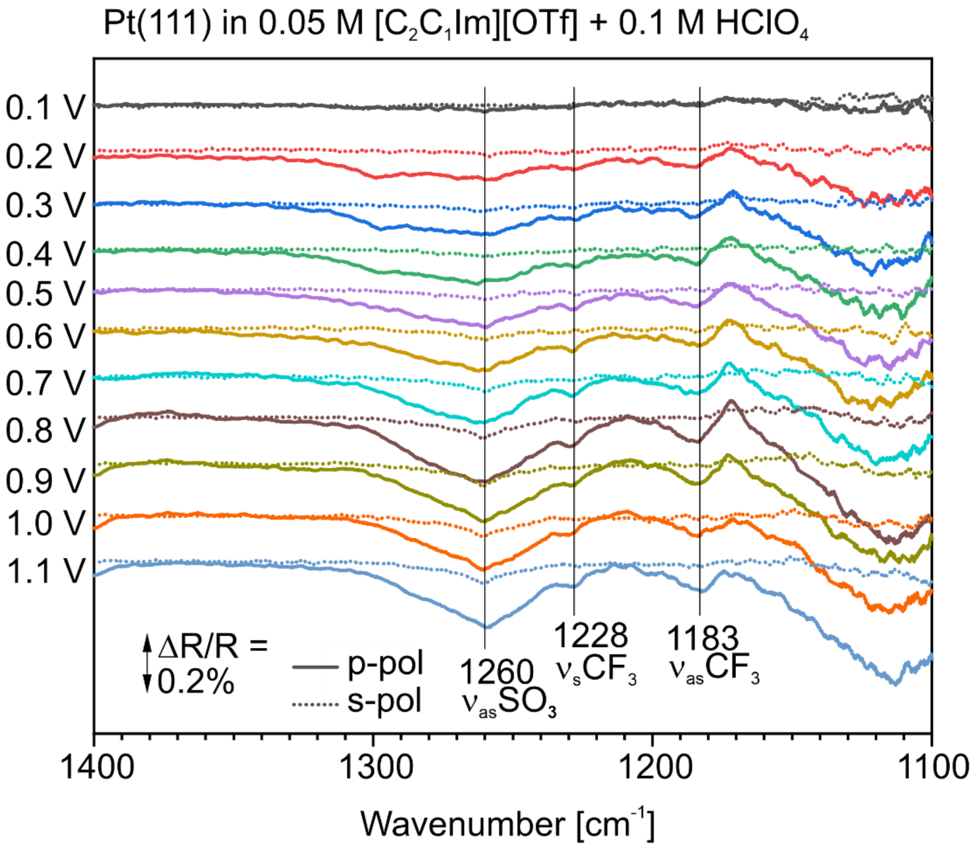

Figure S2. IR spectra measured in a SNIFTIRS experiment in both s- and p-polarization. The sample potentials increased from 0.1 to 1.1 V_{RHE} with a step of 0.1 V. Before each sample potential, the potential was stepped to 0.05 V_{RHE}, while the reference spectra were taken (see **Figure S1**).

2. The IR spectra of 2,3-butanediol, acetoin, and diacetyl

To assign the IR bands of 2,3-butanediol, acetoin, and diacetyl to the different vibrational modes we compared the experimentally obtained IR spectra using attenuated total reflection (ATR) IR spectroscopy with calculated IR spectra obtained by density functional theory (DFT) (see **Figure S3** and **S5**). We summarized the band assignment for 2,3-butanediol, acetoin, and diacetyl in **Table S1**, **S2**, and **S3**, respectively. We visualized the different vibrational modes using the program QVibePlot[2] in **Figure S4**, **S5**, and **S7**.

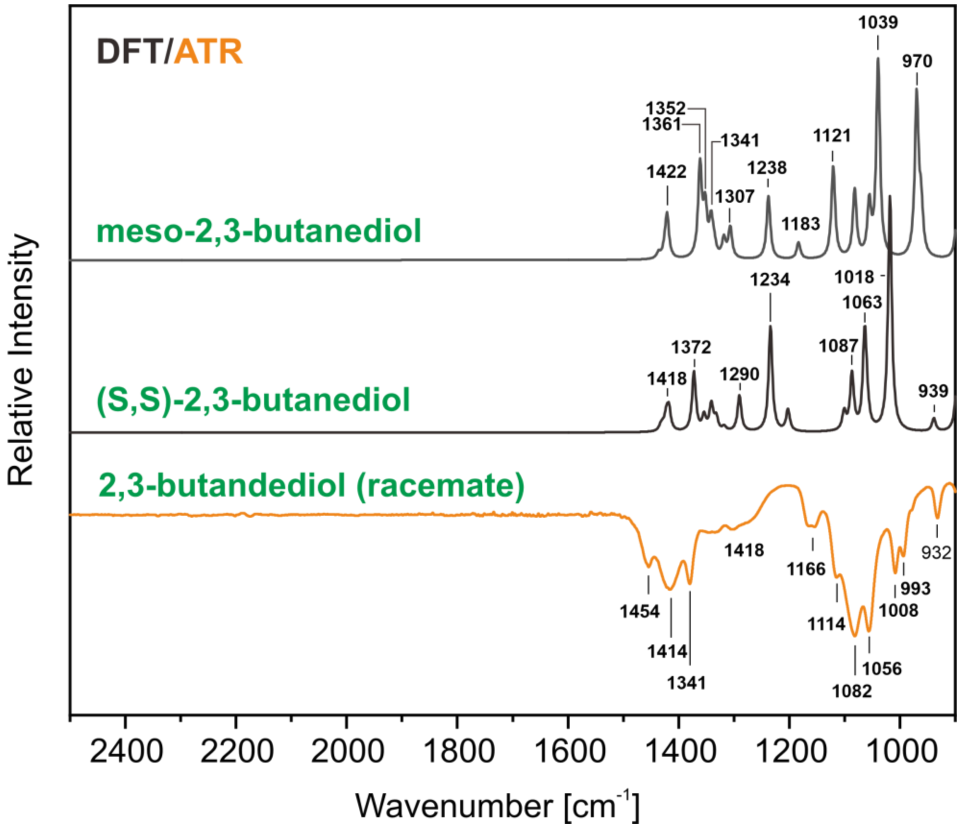

Figure S3: Infrared spectra of 2,3-butanediol in the spectral region from 2500 to 900 cm^{-1}. Simulated spectra of the meso and (S,S) isomers from DFT (black) and experimental IR spectrum of the racemate (orange) recorded with ATR IR spectroscopy. The band assignment is summarized in **Table S1** and the vibrational modes visualized in **Figure S4** and **S5.**

Table S1: Comparison of experimentally observed and theoretically computed vibrational modes of 2,3-butanediol.

ω_{exp} [cm^{-1}]	ω_{DFT} [cm^{-1}]	description
932	939 (SS/RR)	τ(CC)
993	962 (meso)	ν_{as}(C-C-OH)
1008	970 (meso)	ν_{as}(C-C-OH)
1056	1018 (SS/RR)	ν(CC)
1082	1039 (meso)	ν(C-OH)
1114	1055 (meso)<br>1060, **1064** (SS/RR)	ν_{as}(C-C-OH) (meso)<br>ν(CC), ν(CC) (SS/RR)
1154	**1082 (meso)**<br>1087, 1101 (SS/RR)	ν_{as}(CC) (meso)<br>ν(C-OH), ν(C-OH) (SS/RR)
1166	1121 (meso)	ν (CC)
1300*	1183, 1238 (meso)<br>1202, **1234**, 1290 (SS/RR)	δ(COH) δ(COH) (meso)<br>ν(CC), δ(COH), δ(CCC) (SS/RR)

1380	**1307**, 1318 (meso)<br>1318 (SS/RR)	δ(CCH), δ(CCH) (meso)<br>δ(COH) (SS/RR)
1414	1336, 1341, 1352**, 1361** (meso)<br>1332, 1341, 1354 1372 (SS/RR)	ν(CC), ν(CC), δ(COH), ν(CC) (meso)<br>δ(CH_3), δ(CH_3), δ_{sym}(CH_3), ν(CC) (SS/RR)
1454	1420, **1422**, 1426, 1436 (meso)<br>1417, 1422, 1429, 1433 (SS/RR)	δ(CH_3) (meso)<br>δ(CH_3) (SS/RR)

*broad feature with different contributions

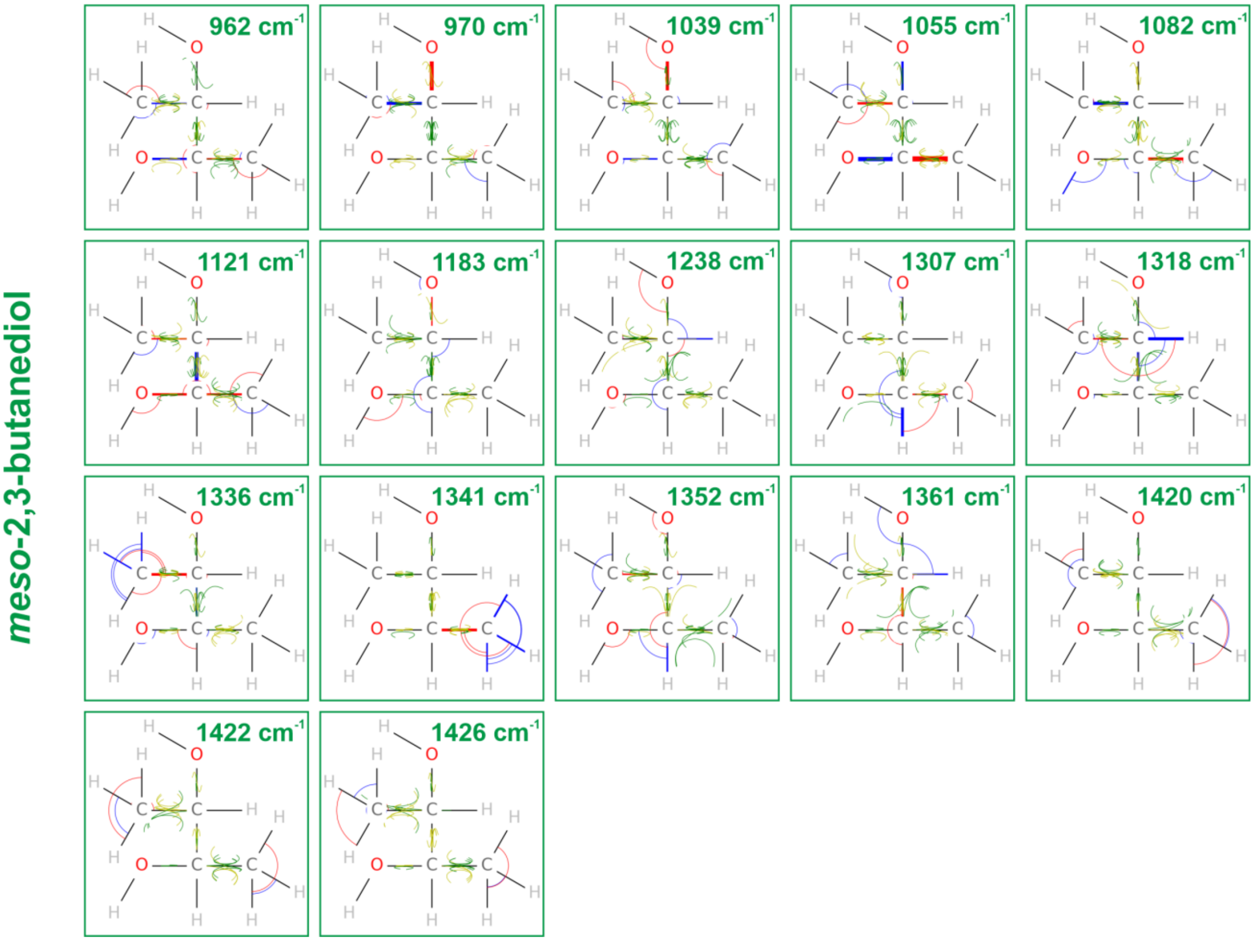

Figure S4: Visualization of the different vibrational modes and the corresponding band positions of the calculated spectra of meso 2,3-butanediol depicted in **Figure S3.**

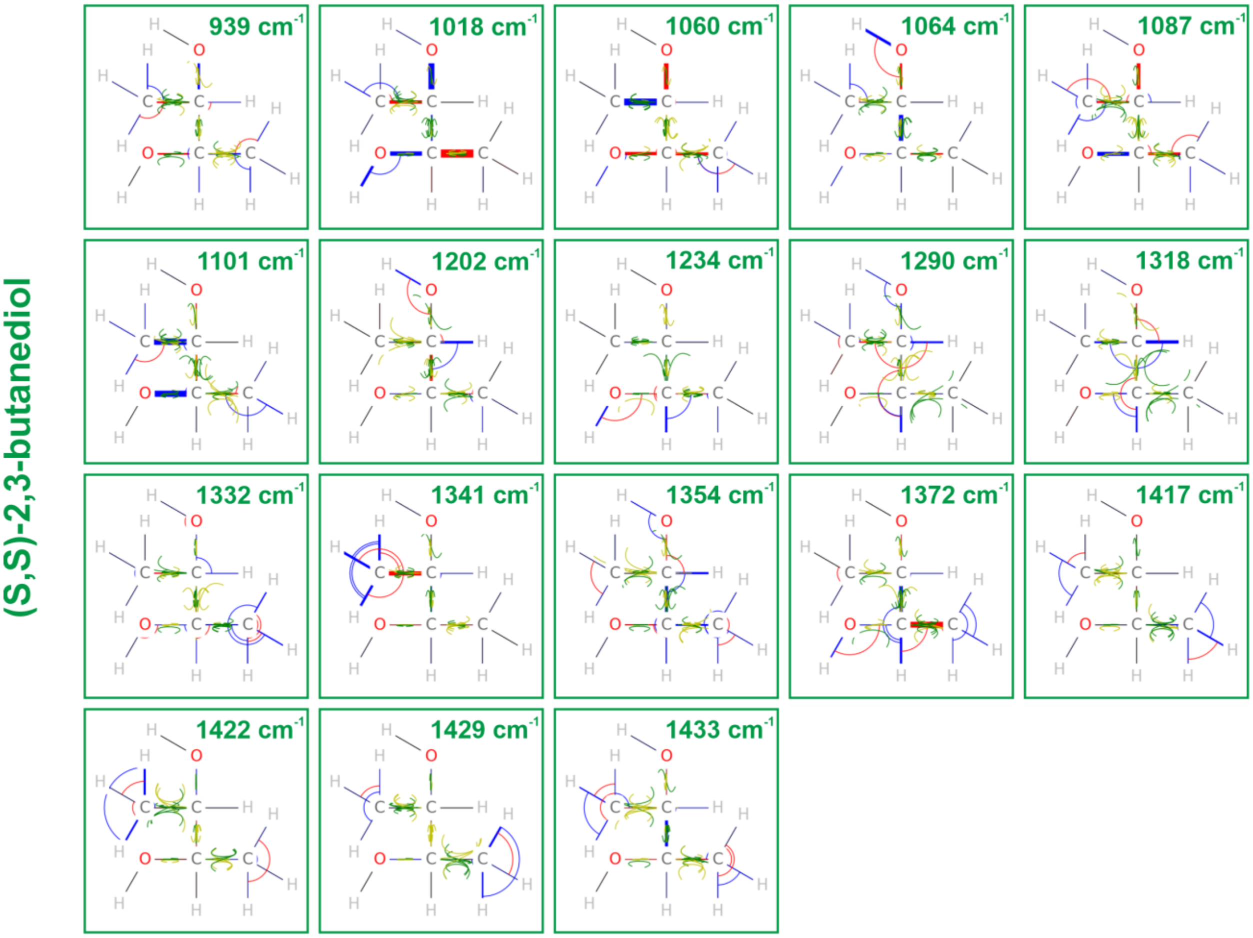

Figure S5: Visualization of the different vibrational modes and the corresponding band positions of the calculated spectra of (S,S)- 2,3-butanediol depicted in **Figure S3.**

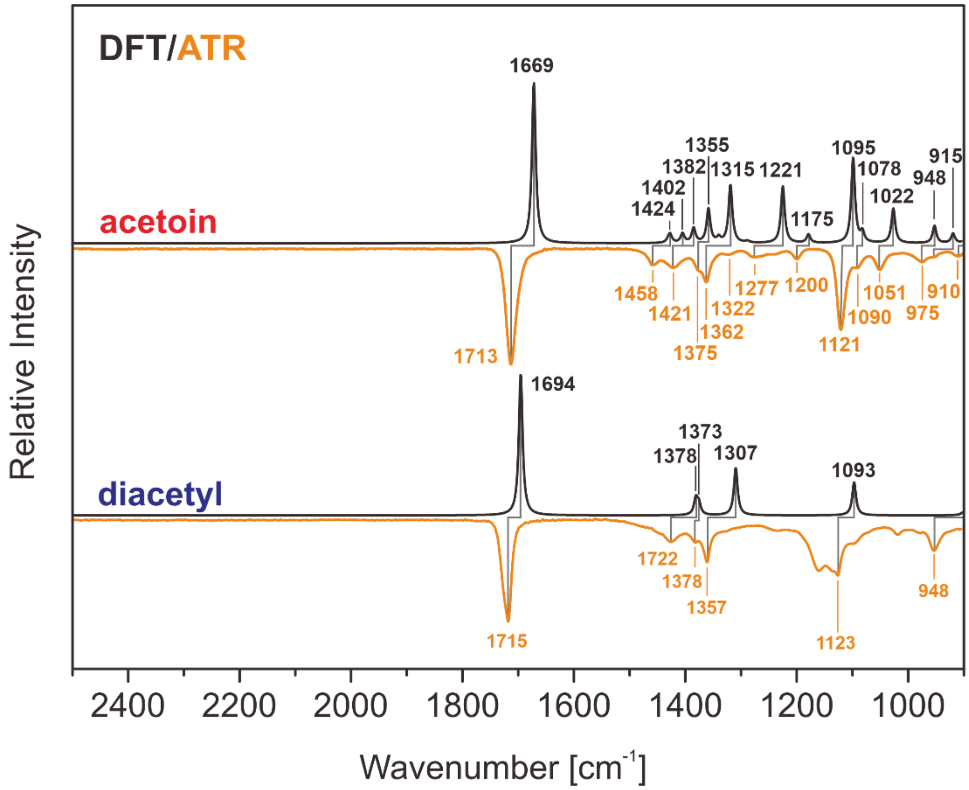

Figure S6: Infrared spectra of the products acetoin and diacetyl in the spectral region from 2500 to 900 cm^{-1}. Simulated spectra from DFT (black) and experimental IR spectrum (orange) recorded in ATR geometry. The band assignment is summarized in **Table S2** (acetoin) and **Table S3** (diacetyl) and the vibrational modes visualized in **Figure S7.**

Table S2: Comparison of experimentally observed and theoretically computed vibrational modes of acetoin.

ω_{exp} [cm^{-1}]	ω_{DTF} [cm^{-1}]	description
910	895	ν(CCOHC)
955	915	δ(CH_3)
975	948	ν(CC)
1051	1022	ν(CC)
1090	1078	ν(CC)
1121	1095	ν(COH)
1200	1175	ν(CC)
1277	1221	ν(CC)
1362	1315	ν(CC)
1375	1355	ν(CC)
1421	**1382**, 1402	δ(CH_3)
1458	1424	δ(CH_3)
1713	1669	ν (C=O)

Table S3: Comparison of experimentally observed and theoretically computed vibrational modes of diacetyl.

ω_{exp} [cm^{-1}]	ω_{DTF} [cm^{-1}]	description
948	885	ν_{as}(CC)
1123	1093	ν_{as}(CC)
1357	1307	ν_{as}(CC)/δ(CH_3)
1378	1373	δ(CH_3)
1422	1378	δ(CH_3)
1715	1694	ν_{as}(C=O)

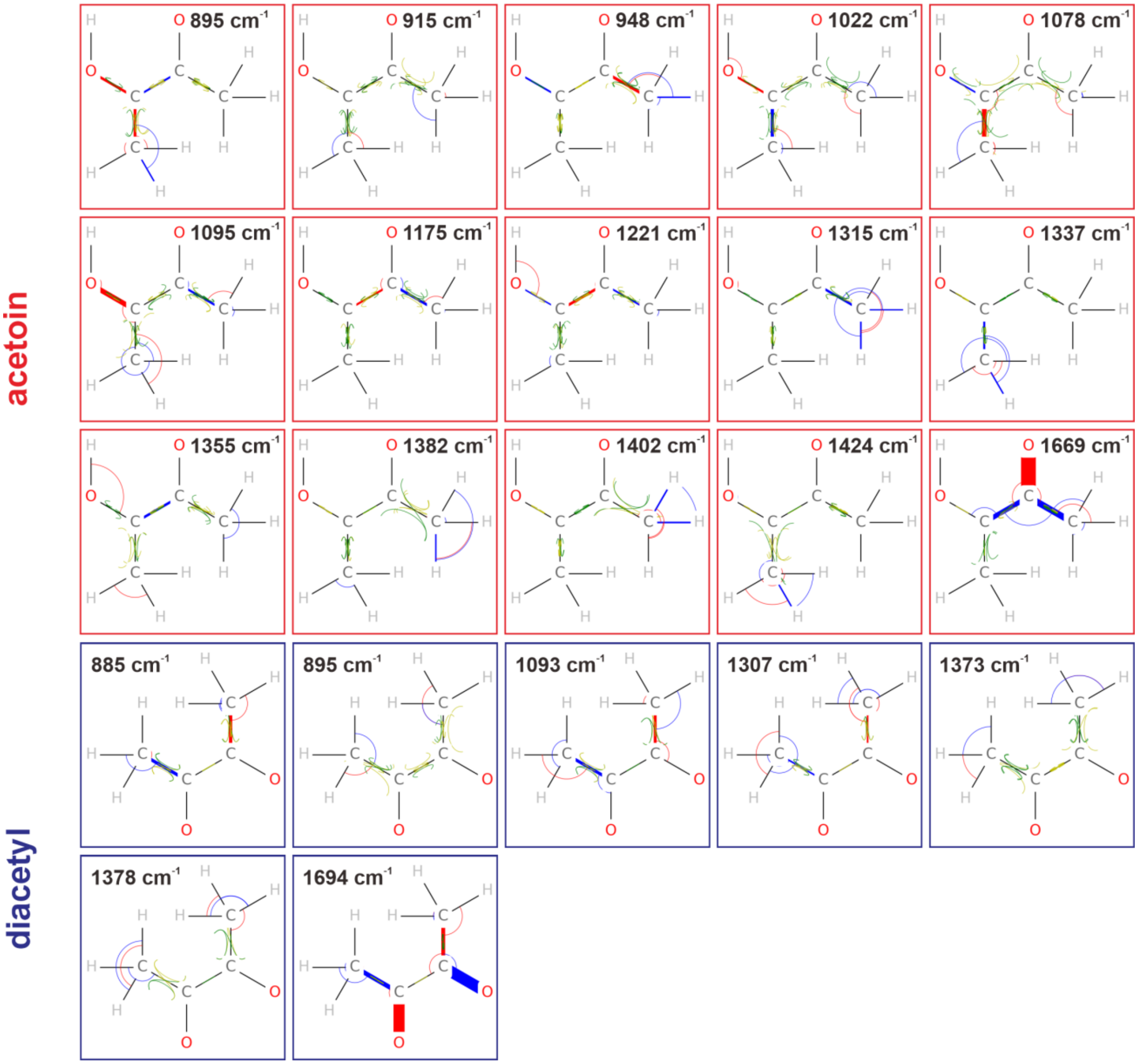

Figure S7: Visualization of the different vibrational modes and the corresponding band positions of the calculated spectra of acetoin and diacetyl depicted in **Figure S6.**

3. EC-IRRA spectra of the 2,3-butanediol oxidation in p-polarization

Figure S8 shows the EC-IRRA spectra of the 2,3-butanediol oxidation measured with p-polarized light. Note that species in solution as well as species adsorbed on the surface are detectable in these spectra. [1] At 0.3 $V_{RHE,}$ we observe a positive band at 1456 cm^{-1} and negative bands at 1713, 1359, and 1200 cm^{-1}. The band at 1200 cm^{-1} forms a maximum at 0.6 V_{RHE} and decreases drastically at higher potentials. At 0.7 V_{RHE} and above a further weak band appears at 2343 cm^{-1}. The positive band at 1457 cm^{-1} we assign to the δ(CH_3) of consumed 2,3-butanediol. The negative bands at 1713, 1359 cm^{-1} are referred to the ν(CO) and ν(CC) vibrations of acetoin and diacetyl. As the band at 1200 cm^{-1} is a specific marker for the intermediate acetoin, we conclude that acetoin is initially formed as an intermediate with an onset potential at 0.3 V_{RHE} and reaches highest concentration at 0.6 $V_{RHE,}$ while at higher potentials acetoin is further oxidized to diacetyl and the concentration decreases rapidly. The results measured with p-polarized light confirm the results measured with s-polarized light (**Figure 2**, main text). Finally, no adsorbed CO_{ads} is observable in the spectral region between 1800 and 2100 cm^{-1}. This indicates that no CC bond splitting occurs on the Pt(111) surface and no CO_{ads} is formed during the reaction. A similar behavior we observed also for other secondary alcohols as isopropanol.[3]

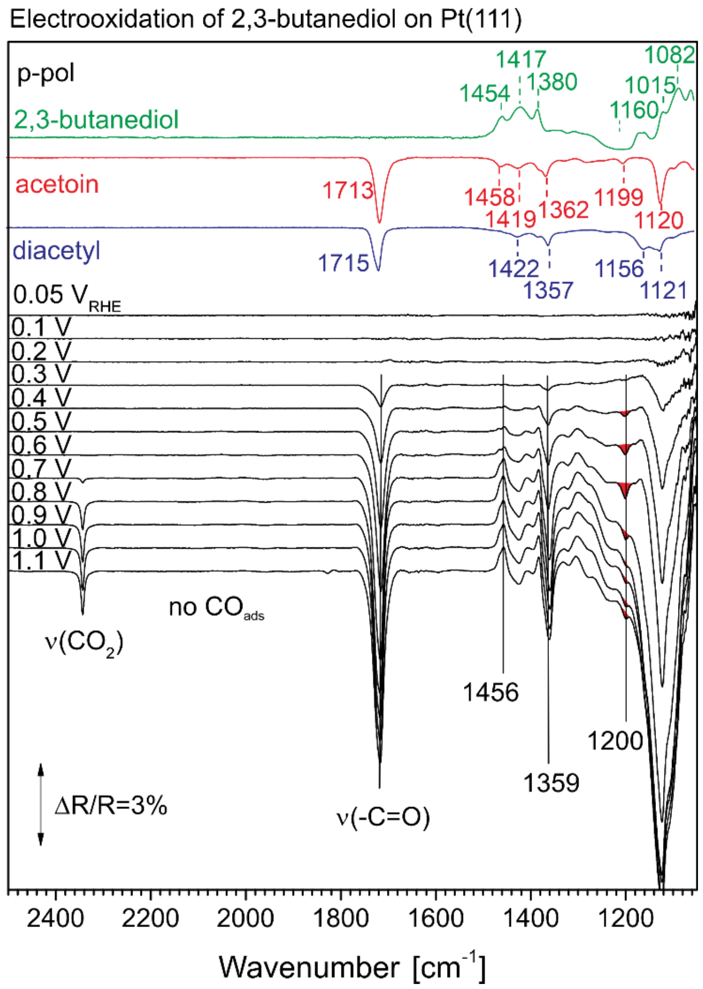

Figure S8: EC-IRRA spectra of the oxidation of 2,3-butanediol on Pt(111); (a) reference spectra of 2,3-butanediol, acetoin, and diacetyl measured with ATR; (b) in-situ IR spectra. The spectra were measured with p-polarized light and the reference spectrum was taken at 0.05 V_{RHE}.

4. Quantification of IR bands

We analyzed the spectra shown in **Figure 2** (main text) quantitatively using the approach described in **Section 5** of the **Supporting Information**. **Figure S9a** shows the potential dependent concentration of 2,3-butanediol (green), acetoin (red), diacetyl (blue) as well as the sum of the products acetoin and diacety (grey) in the absence of the IL [C_2C_1Im][OTf]. The onset of the 2,3-butanediol oxidation occurs at 0.3 V_{RHE} forming initially acetoin. The onset potential of diacetyl formation is 0.5 $V_{RHE.}$ The concentration of acetoin reaches a maximum at 0.6 V_{RHE} and decreases at higher potentials rapidly, indicating the oxidation of acetoin forming diacetyl. At potentials ≥0.8 V_{RHE} the 2,3-butanediol oxidation stops due the formation of a passivating hydroxide layer. Note that we assign changes in the concentration at potentials above 0.8 V_{RHE} to diffusion between the thin layer and the bulk solution. In the presence of the IL (**Figure S9b**) only a small fraction of diacetyl is formed and the oxidation of acetoin is sufficiently suppressed.

.

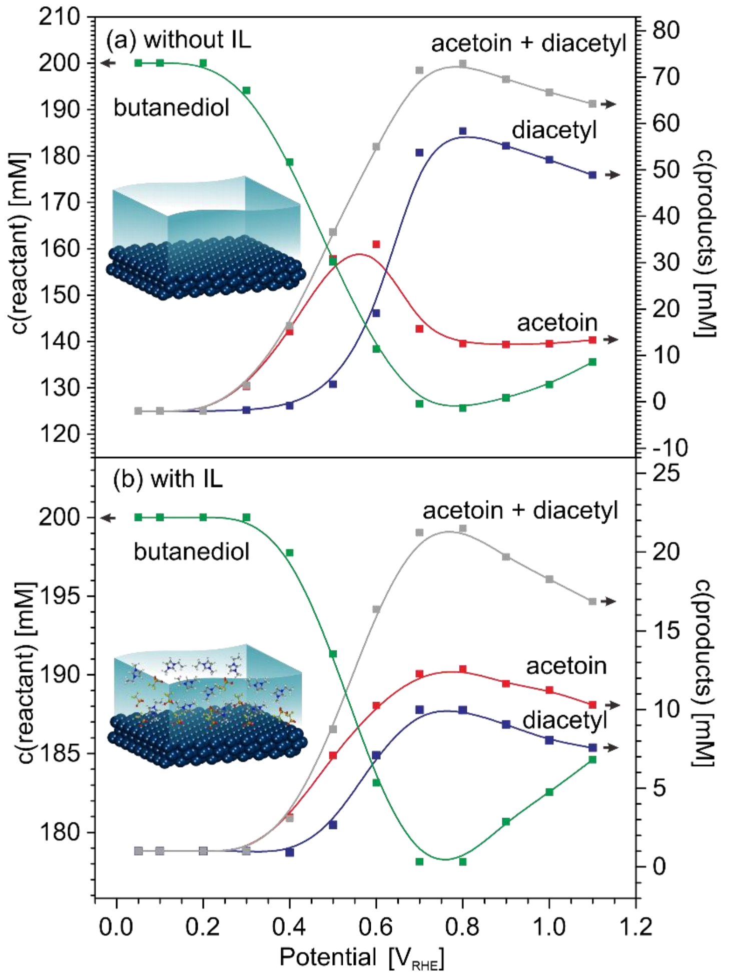

Figure S9: Potential dependent development of the concentrations of 2,3-butanediol (green), acetoin (red), diacetyl (blue) and acetoin + diacetyl (grey) in the (a) absence and (b) presence of the IL [C_2C_1Im][OTf].

5. Description of the quantitative analysis

a) Determination of the extinction coefficients

To determination the extinction coefficients, a dilution series of 2,3-butanediol, acetoin, and diacetyl with different concentrations *c* was prepared, and the absorbance A_λ was measured using a transmission cell with a layer thickness d of 50 µm (see **Figure S10**). The extinction coefficients ε_λ were determined from linear fits of the Beer Lambert law (see **Figure S11**):

$$A_\lambda = \varepsilon_\lambda \cdot d \cdot c$$

The obtained extinction coefficients are given in **Table S4**.

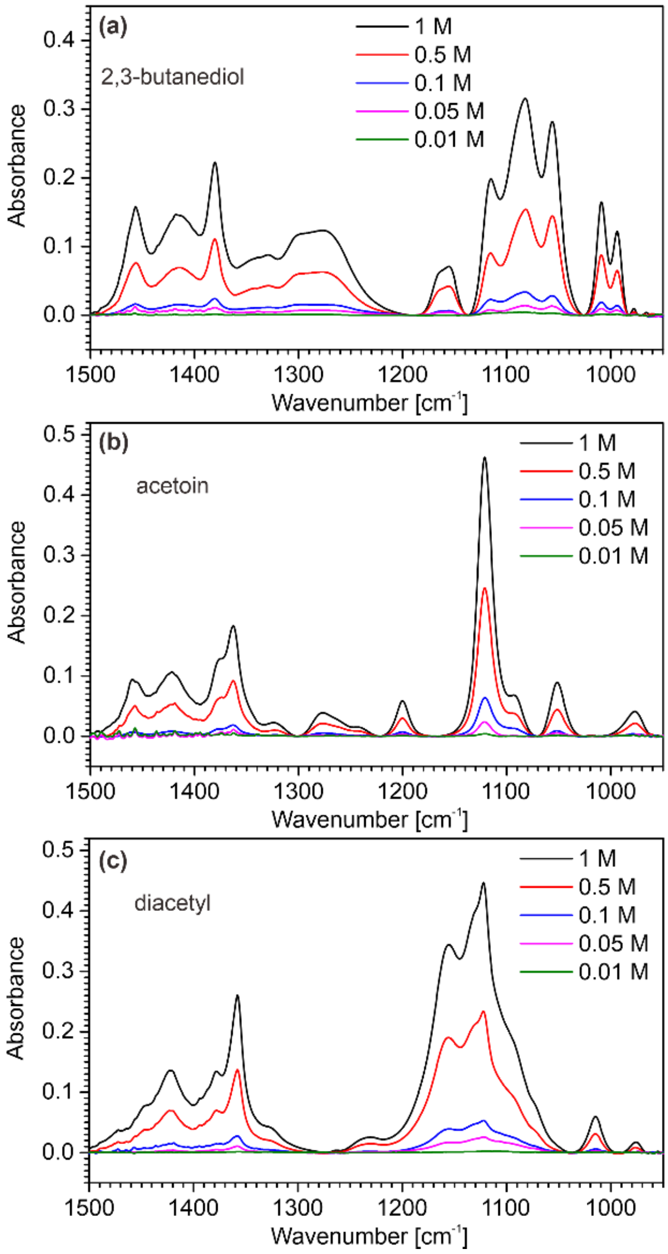

Figure S10: Transmission IR spectra of a serial dilution experiment of (a) 2,3-butanediol, (b) acetoin, and (c) diacetyl. The concentrations of chemicals were in the range of 0.01–1 M and the layer thickness was 50 μm.

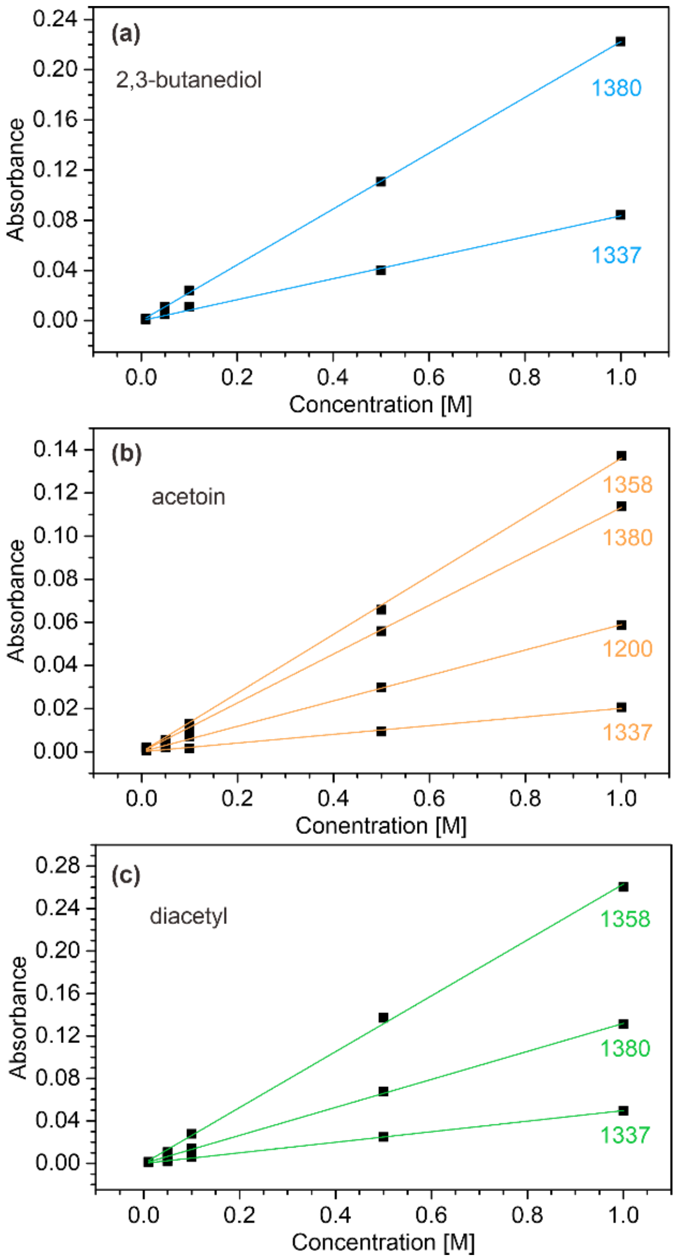

Figure S11: Absorbance as a function of the concentration for the IR bands of (a) 2,3-butanediol, (b) acetoin, and (c) diacetyl used for quantification. The transmission spectra were measured with a layer thickness of 50 μm.

Table S4: Band positions used for quantification and corresponding extinction coefficients determined for 2,3-butanediol, acetoin, and diacetyl.

ν_{exp} [cm^{-1}]	species	ε_{exp} [L·mol^{-1}·cm^{-1}]
1200	acetoin	12
1337	butanediol	17
	acetoin	4.0
	diacetyl	9.9
1358	acetoin	27
	diacetyl	53
1380	butanediol	44
	acetoin	23
	diacetyl	26

b) Determination of the average beam path in the thin film

We determined the average beam path of the IR beam in the thin layer using the Beer Lambert law. The absorbance was determined from the single channel spectra of 2,3-butanediol at 0.05 V_{RHE}. Using the absorbance of the IR band of 2,3-butanediol at 1380 cm^{-1} and the extinction coefficient given in **Table S4**, we calculated the average beam path d of the IR beam in the thin layer:

Table S5: Calculated average beam path d of the IR beam in the thin layer.

	Band [cm^{-1}]	ε [L·mol^{-1}·cm^{-1}]	Δc [mol·L^{-1}]	A	d [μm]
Experiments without IL	1380	44	0.2	0.02971	34
Experiments with IL	1380	44	0.2	0.03442	39

c) Calculation of the concentration change of 2,3-butanediol, acetoin, and diacetyl

Using the determined thin layer thickness and the extinction coefficients of selected IR bands, we calculated the change of concentration of 2,3-butanediol, acetoin, and diacetyl as a function of potential.

First, we calculated the change of concentration of acetoin using the IR band of acetoin at 1200 cm^{-1}, which does not overlap with bands of 2,3-butanediol and diacetyl. The resulting data are displayed in **Figure S12**.

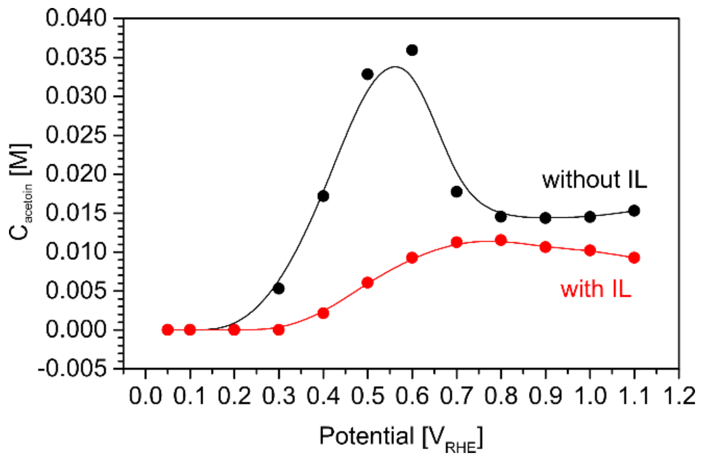

Figure S12: Concentration changes of acetoin as a function of potential in experiments with IL (red curve) and without IL (black curve).

To determine the change of concentration of diacetyl, we use the difference in absorbance ΔA between 1358 and 1337 cm^{-1}. Measuring the difference enables us to be independent of the background. However, ΔA of diacetyl ($\Delta A_{diacetyl\_1358}$) overlaps with ΔA of acetoin ($\Delta A_{acetoin\_1358}$) (see **Figure S13**). Therefore, we calculated $\Delta A_{diacetyl\_1358}$ by subtracting $\Delta A_{diacetyl\_1358}$ from total ΔA (ΔA_{total_1358}):

$$\Delta A_{diacetyl\_1358} = \Delta A_{total\_1358} - \Delta A_{acetoin\_1358}$$

Note that the difference in the extinction coefficients $\Delta\varepsilon_{1358}$ of 2,3-butanediol between 1358 and 1337 cm^{-1} is very small. Consequently, we did not take into account changes in the $c_{2,3\text{-butanediol}}$ for the determination of $c_{diacetyl}$.

Using the Beer Lambert law, we obtained:

$$\Delta A = \Delta\varepsilon \cdot d \cdot c \; \Delta\varepsilon_{diacetyl\_1358} \cdot d \cdot c_{diacetyl} = \Delta A_{total\_1358} - \Delta\varepsilon_{acetoin\_1358} \cdot d \cdot c_{acetoin}$$

Using the determined thin layer thickness and the extinction coefficients, we calculated the concentration change of diacetyl. The result is shown in **Figure S14**.

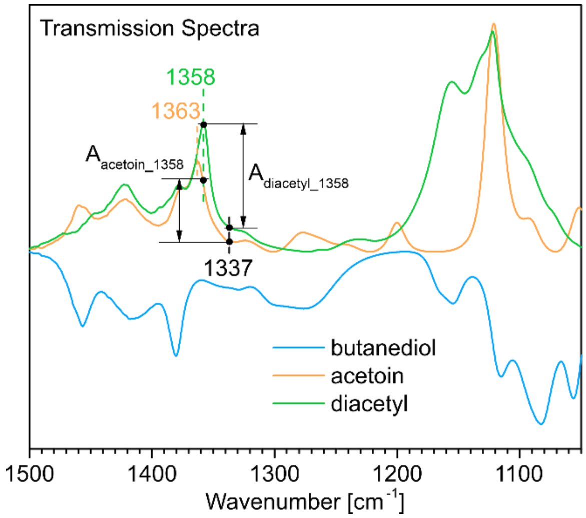

Figure S13: Transmission IR spectra of 2,3-butanediol (blue curve), acetoin (orange curve), and diacetyl (green curve) with a layer thickness of 50 µm; illustration of absorbance of acetoin and diacetyl at 1358 cm^{-1}.

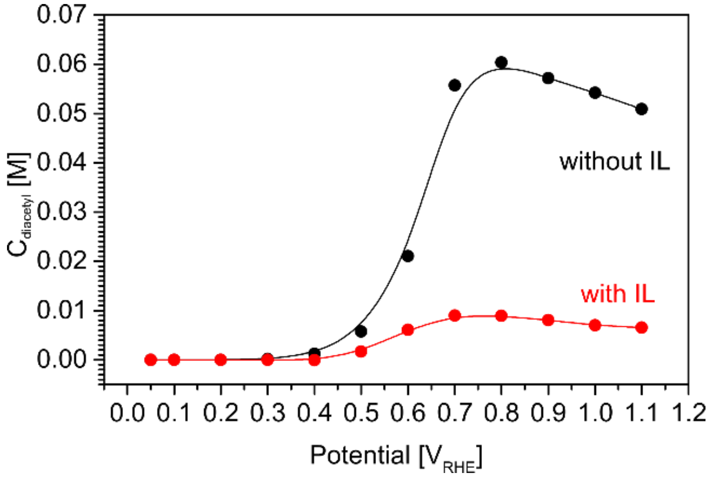

Figure S14: Concentration changes of diacetyl as a function of potential in experiments with IL(red curve) and without IL (black curve).

To determine the change of concentration of 2,3-butanediol, we use ΔA of 2,3 butanediol ($\Delta A_{butanediol\_1380}$) between 1380 and 1337 cm^{-1}. However, $\Delta A_{butanediol\_1380}$ overlaps with ΔA of acetoin and diacetyl (see **Figure S15**). Therefore, we calculated $\Delta A_{butanediol\_1380}$ from ΔA_{total_1380}, $\Delta A_{acetoin\_1380}$, and $\Delta A_{diacetyl\_1380}$:

$$\Delta A_{butanediol\_1380} = \Delta A_{total\_1380} + \Delta A_{acetoin\_1380} + \Delta A_{diacetyl\_1380}$$

Using the Beer Lambert law, we obtained:

$$(\Delta \varepsilon_{butanediol\_1380}) \cdot d \cdot c_{butanediol} = \Delta A_{total\_1380} + (\Delta \varepsilon_{acetoin\_1380}) \cdot d \cdot c_{acetoin} + (\Delta \varepsilon_{diacetyl\_1380}) \cdot d \cdot c_{diacetyl}$$

Using the determined thin layer thickness and the extinction coefficients, we calculated the concentration change of 2,3-butanediol. The result is shown in **Figure S16**.

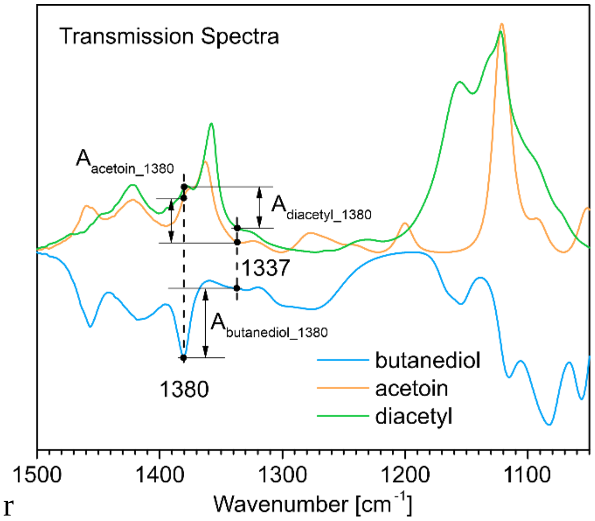

r

Figure S15: Transmission IR spectra of 2,3-butanediol (blue curve), acetoin (orange curve), and diacetyl (green curve) with a layer thickness of 50 µm, and the illustration of ΔA_{1380} of 2,3-butanediol, acetoin, and diacetyl.

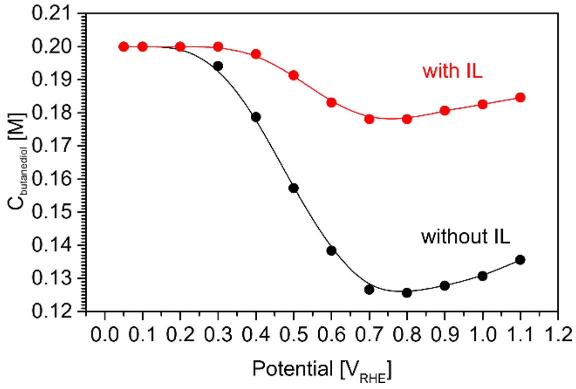

Figure S16: Concentration changes of 2,3-butanediol as a function of potential in experiments with IL(red curve) and without IL (black curve).

The selectivity of acetoin was determined by the following equation:

$$\text{Selectivity}_{\text{acetoin}} = \frac{C_{acetoin}}{C_{acetoin}+C_{diacetyl}}$$

The calculated selectivity of acetoin in both experiments is shown in **Figure 3** in the main text.

6. Experimental part

Cleaning procedure

To avoid contaminations from the equipment, we cleaned all glass and Teflon parts as well as all noble metal wires with a procedure described in the following. We stored all parts in a solution of NoChromix (Sigma Aldrich) and H_2SO_4 (Merck, EMSURE, 98%) overnight. Afterwards, we rinsed the equipment at least five times with ultra-pure water (Milli-Q Synergy UV, 12.2 MΩ·cm at 25 °C, TOC < 5 ppm) and performed 3 boiling and rinsing cycles in ultra-pure water. We annealed all noble metal wires in the flame of a Bunsen burner and rinsed the wires subsequently with ultra-pure water.

Preparation of Pt(111) and solutions

We annealed Pt(111) single crystals (MaTecK, 99.999%, depth of roughness <0.01 µm, accuracy of orientation <0.4°) in the blue flame of a Bunsen burner for 2 min. Subsequently we cooled the single crystals in Ar/H_2 (Linde 5.0; volume ratio of ~3:1) atmosphere to room temperature. For the transfer to the electrochemical cell we protected the surface of the single crystals with a droplet of ultrapure water. We prepared solutions from 2,3-butanediol (Sigma Aldrich 98%), acetoin (Sigma Aldrich 98%), diacetyl (Sigma Aldrich, 97%), and $HClO_4$ (Merck, Suprapur). To guarantee highest purity, the IL [C_2C_1Im][OTf] was synthesized and purified in-house following a procedure described previously.[4] We degassed all solutions by purging with Ar (Linde, 5.0) for at least 20 min.

EC-IRRAS measurements

To measure electrochemical infrared reflection absorption spectroscopy (EC-IRRAS) we used commercial IR spectrometers with evacuated optics (Bruker Vertex 80v) and liquid nitrogen cooled mercury cadmium telluride (MCT) narrow band detectors. The spectrometers are equipped with commercial optics for electrochemical measurements, home-build electrochemical cells and polarizers. We used CaF_2 hemispheres (Korth) as IR transparent window material sealed by Kalrez gaskets. We controlled the potential by commercial potentiostats (Gamry Reference 600 or Reference 600+). We used Pt wires as counter electrodes and home build or commercial (Mini-HydroFlex, Gaskatel) reversible hydrogen electrodes (RHE) as reference electrodes. We recorded reference spectra (256 scans per spectrum) at 0.05 V_{RHE} before each measurement. We took IR spectra with 128 scans per spectrum, a resolution of 2 cm^{-1}, and an acquisition time of 57 s per spectrum between 0.05 and 1.1 V_{RHE}. The spectra were measured with p- and s-polarized light, respectively. For a more detailed description of the used IR setups, we refer to literature.[5]

Density functional theory

We performed gas-phase density functional theory (DFT) calculations with the Turbomole Software package version 7.2.[6] The exchange-correlation functional PBE by Perdew, Burke and Ernzerhof[7] was used with the def2-TZVP basis set by Weigend and Ahlrichs[8]. The RI-J approximation[8b] was applied to accelerate the calculations. Long range dispersion interactions were included by using the D3 dispersion correction scheme.[9] The COSMO solvation model[10]

was applied, using a dielectric constant of 80. Harmonic frequency calculations were performed with analytical gradients to obtain the vibrational spectra.

References

[1] T. Iwasita, F. C. Nart, *Progress in Surface Science* **1997**, *55*, 271-340.
[2] M. Laurin, *Journal of Chemical Education* **2013**, *90*, 944-946.
[3] F. Waidhas, S. Haschke, P. Khanipour, L. Fromm, A. Görling, J. Bachmann, I. Katsounaros, K. J. J. Mayrhofer, O. Brummel, J. Libuda, *ACS Catalysis* **2020**, *10*, 6831-6842.
[4] O. Brummel, F. Faisal, T. Bauer, K. Pohako-Esko, P. Wasserscheid, J. Libuda, *Electrochimica Acta* **2016**, *188*, 825-836.
[5] F. Faisal, M. Bertram, C. Stumm, F. Waidhas, O. Brummel, J. Libuda, *Review of Scientific Instruments* **2018**, *89*, 114101.
[6] TURBOMOLE V7.2 2017, a development of University of Karlsruhe and Forschungszentrum Karlsruhe GmbH, 1989-2007, TURBOMOLE GmbH, since 2007; available from http://www.turbomole.com.
[7] J. P. Perdew, K. Burke, M. Ernzerhof, *Physical Review Letters* **1996**, *77*, 3865-3868.
[8] a) F. Weigend, R. Ahlrichs, *Physical Chemistry Chemical Physics* **2005**, *7*, 3297-3305; b) F. Weigend, *Physical Chemistry Chemical Physics* **2006**, *8*, 1057-1065.
[9] S. Ehrlich, J. Moellmann, W. Reckien, T. Bredow, S. Grimme, *ChemPhysChem* **2011**, *12*, 3414-3420.
[10] A. Schäfer, A. Klamt, D. Sattel, J. C. W. Lohrenz, F. Eckert, *Physical Chemistry Chemical Physics* **2000**, *2*, 2187-2193.
